# Prevalence of polycystic ovary syndrome and its associated complications in Iranian women: A meta-analysis

**Published:** 2015-10

**Authors:** Anahita Jalilian, Faezeh Kiani, Fatemeh Sayehmiri, Kourosh Sayehmiri, Zahra Khodaee, Malihe Akbari

**Affiliations:** 1 *Department of Obstetrics and Gynecology, School of Medicine, Ilam University of Medical Sciences, Ilam, Iran.*; 2 *Student Research Committee, Ilam University of Medical Sciences, Ilam, Iran.*; 3 *Department of Social Medicine, School of Medicine, Ilam University of Medical Sciences, Ilam, Iran.*; 4 *Department of Nursing and Midwifery, Ilam University of Medical Sciences, Ilam, Iran.*

**Keywords:** Polycystic ovary syndrome, Prevalence, Hirsutism, Acne, Obesity

## Abstract

**Background:**

Polycystic ovary syndrome (PCOS) is the most common endocrine disorder in women of reproductive age and is the most common cause of infertility due to anovulation. There is no single criterion for the diagnosis of this syndrome.

**Objective:**

The purpose of this study was to investigate the prevalence of PCOS and its associated complications in Iranian women using meta-analysis method.

**Materials and Methods:**

Prevalence of PCOS was investigated from the SID, Goggle scholar, PubMed, Magiran, Irandoc, and Iranmedex, and weighting of each study was calculated according to sample size and prevalence of the binomial distribution. Data were analyzed using a random-effects model meta-analysis (Random effects model) and the software R and Stata Version 11.2.

**Results:**

30 studies conducted between the years 2006 to 2011 were entered into meta-analysis. The total sample size was 19, 226 women aged between 10-45 years. The prevalence of PCOS based on National institute of child health and human disease of the U.S was, 6.8% (95 % CI: 4.11–8.5), based on Rotterdam was 19.5% (95 % CI: 2.24-8.14), and based on ultrasound was 4.41% (95% CI: 5.68-4.14). Also, the prevalence of hirsutism was estimated to be 13%, acne 26%, androgenic alopecia 9%, menstrual disorders 28%, overweight 21%, obesity 19%, and infertility 8%.

**Conclusion:**

The prevalence of PCOS in Iran is not high. However, given the risk of complications such as heart disease - cardiovascular and infertility, prevention of PCOS is important; we suggest that health officials must submit plans for the community in this respect.

## Introduction

Polycystic ovary syndrome (PCOS) is the most common endocrine disorder in women of reproductive age and is the most common cause of infertility due to anovulation. Various studies have reported a prevalence of 5-10% for PCOS, for the first time in 1935, the classic form of PCOS was described by Ashtyn and Leventhal (1). It appears that several factors may be involved in its development. Perhaps the disease exists as a genetic predisposition in the person and its symptoms are exacerbated by environmental factors and lifestyle (2). PCOS symptoms involve both endocrine and gynecologic system; as amenorrhea or oligo amenorrhoea, hirsutism, obesity, acne, androgenic alopecia and reproductive disorders (3). PCOS is not a disease exclusive to fertility and adolescence period; rather it can be associated with varying effects on a person's life. The main complications of the disease in adolescence are the incidence of amenorrhea, oligo menorrhea, hirsutism, obesity, and acne. In fertility ages, the patient’s chief complaint is infertility and irregular ovulation. The complications of adolescence ages still exist in this period. In pre-menopausal and post-menopausal ages, this syndrome can increase the risk of type 2 diabetes, hypertension, dyslipidemia, cardiovascular diseases and even endometrial cancer and possibly breast cancer (4). In total, 30 to 40 percent of women with PCOS have experienced impaired glucose tolerance or diabetes before age 40. Patients with PCOS are at risk for a group of metabolic disorders including insulin resistance, glucose intolerance impairment, diabetes, hypertension, lipid disorders, cardiovascular disease, and increased risk of endometrial, uterine, and breast cancers. PCOS is a heterogeneous disorder in which both increased ovarian androgens and possibly adrenal and some degree of metabolic disorders exist (2).

Two principal components to diagnose this syndrome are menstrual dysfunction and clinical or laboratory hyperandrogenism in which these items are used in clinical diagnosis (5). Most patients with PCOS may only show one or two clinical symptoms. The most common clinical finding is menstrual disorders which is usually started from menarche, or immediately after it and may appear in the form of oligo menorrhea, amenorrhea or poly menorrhea and might even being normal menstrual cycle (6, 7). Clinical hyperandrogenism symptoms include symptoms such as hirsutism, acne, androgenic alopecia, and incidence of male characteristics which occurs in 66% of adolescents with PCOS (8).

PCOS is usually associated with hormonal abnormalities through changes in the concentrations of luteinizing hormone (LH), prolactin, estrogen and serum androgens (testosterone and androstenedione). Hormonal measurements indicate that many women with PCOS have increased LH/FSH ratio. Therefore, the ratio of 2 to 1 and sometimes 2.5 was considered as a measure of biochemical disease (7). Sequence of events that eventually leads to hyperandrogenism, as the symptoms of the disease, abnormal pulsatile pattern of LH and menstrual disorders might be triggered by a variety of different body parts and processes. The disease may begin by dysfunction in adrenal function, hypothalamic or central nervous system or just the ovary. It seems that the prevalence of polycystic ovaries is higher among women younger than 35 years. The prevalence of this syndrome has been estimated among different populations in various studies (9). PCOS prevalence has been reported between 2.2% to 26% in different countries depending on various population studies, the criteria used to define it, and the methods used to define any criteria (10). There is no single criterion for the diagnosis of this syndrome. Rather, it is diagnosed based on a combination of the results of clinical, laboratory and ovarian morphology in ultrasound, but three definitions are often used for its diagnosis (4). The first definition was proposed by the National Institute of Health (NIH) in 1990 in which clinical and biochemical signs of hyperandrogenism or hyperandrogenemia and clinical symptoms of ovulation disorder as amenorrhea, oligomenorrhea or infertility in the absence of non-classical adrenal hyperplasia are the diagnostic criteria of the disease (8, 9). The second definition (Rotterdam) was presented by Fertility and Embryology Association of Europe and America Fertility Society in Rotterdam conference in 2003 and has considered two criteria from the following three criteria as criteria for diagnosis of PCOS (11):

Oligoovulation: menstrual period more than 40 days or anovulation less than 9 cycles per year.

Clinical hyperandrogenism: (acne, hirsutism, and androgenic alopecia) or biochemical hyperandrogenism (elevated serum androgen levels).

The presence of polycystic ovaries on pelvic ultrasound: (more than 12 follicles measuring 2 to 9 mm and ovarian volume greater than 10 mm) (12, 11).

The third and the newest definition was proposed in 2006 by Androgen Excess Society (AES) that has considered the following criteria for the diagnosis of PCOS.

1. Hirsutism or hyperandrogenism

2. Oligoovulation and anovulation or polycystic ovaries

3. Increase level of androgens or related disorders (9). In some studies, PCOS is only diagnosed based on ultrasound procedures. Among sonographic findings, the basal follicle counts by vaginal ultrasonography can be mentioned in the diagnosis of PCOS in which basal follicles more than 10 is considered as one of the criteria for PCOS. Echogenicity increased stroma, stromal hypertrophy, increased volume and size of ovarian and measuring the ratio of stroma to the total surface area of ovarian are the other factors (4). Therefore, given the impact of PCOS on the incidence of many disorders, the present study aims at investigating the prevalence of this syndrome and its associated symptoms among Iranian women using meta-analysis method.

## Materials and methods

This paper was written by PRISMA guideline. To decrease bias, two authors (Sayehmiri F, kiani F) searched, selected papers, and extracted data of paper independently.

The findings of this study were based on all articles published in the national and international journals and student's thesis. All national scientific databases (Iranmedex, SID, Magiran, Irandoc, and Medlib) and international databases (PubMed/Medline, Scopus, and ISI Web of Knowledge. After reading the summary of the individual papers, irrelevant articles were rejected and possibly related papers were identified to be fully studied. All cross sectional and review studies in relation to the prevalence of PCOS were investigated regardless of the time of publication. Papers were selected through searching Persian and English keywords such as polycystic ovary syndrome, prevalence, women, hirsutism, acne, alopecia, menstrual disorders, obesity, infertility, and a meta-analysis with all the possible combinations and keywords.

**Study selection and data extraction**

**Figure 1:**
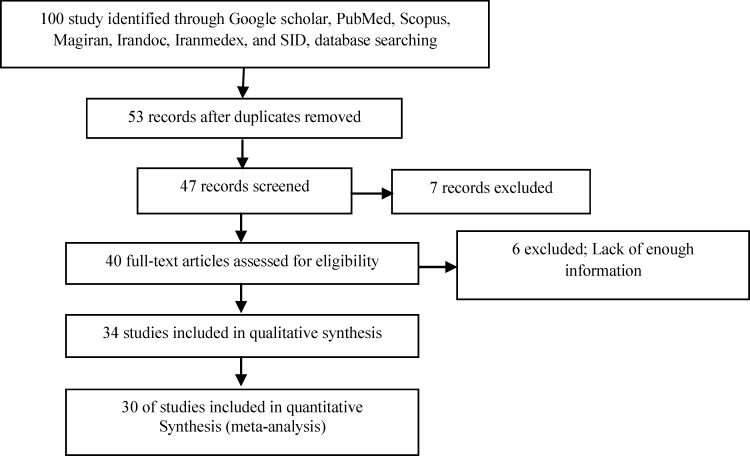
The flowchart of selected articles for final analysis

The main criterion for entering various articles to this research was the prevalence of PCOS among Iranian women. Also, the prevalence of PCOS was reviewed in a number of relevant articles with different criteria and failure to report the same results in other papers. Article associated with each of these disorders were also found, and then were checked to enter the analysis if they are relevant. Studies which were not among the first investigations and irrelevant studies (studies done in special subgroups) were excluded from the study. First, the researcher collected all articles related to PCOS and after completion of the search provided a list of abstracts. At this stage, all the articles with title keywords of "frequency" and "polycystic ovary syndrome" were entered into the initial list. Later, a check list of information required for all studies being initially evaluated were provided for the final evaluation. Accordingly, this study selected 31 articles in which random sampling was used in all of them to determine the sample were studied. Next, a form consisting of information needed for the study (investigator's name, title, year, location, number of samples, the overall prevalence based on various criteria, the prevalence of associated complications, the mean age of patients and body mass index) was designed. The underlying data needed for this study were entered into the chart to be analyzed. Studies with sample of patients with PCOS only in Iran and the use of standardized assessment tools were finally confirmed. Finally, 30 suitable articles were included to the meta-analysis stage (Figure 1). No meta-analysis study was found in Iran on the prevalence of PCOS. Full texts of articles were reviewed for analysis. 

**Statistical analysis**

Since the prevalence of PCOS and the sample number have been extracted in each study, the binomial distribution was used to calculate the variance of each study. The average weight was used to combine the prevalence of various studies. Each study was weighted in proportion to its variance. Due to large differences in prevalence of different studies (heterogeneity of studies) and the significance of the heterogeneity index (I^2^), the random effects model was used in the meta-analysis.

## Results

In the present study, 30 reports and articles related to the topic, that were carried out between 2006 and 2011 were used for conducting meta-analysis and systematic review. The total sample size included 19,226 women aged between 18-44 years. Degree of heterogeneity was investigated (heterogeneity index) in the level of prevalence among the different findings of studies. The random effects model was used in all subsequent steps due to the high heterogeneity of the results of studies (Table I).

Systematic review and meta-analysis results in a separate section to determine the "prevalence of PCOS according to NIH, Rotterdam , and ultrasound techniques, and determining the prevalence of associated complications is provided: The prevalence of PCOS based on NIH, Rotterdam standards, and ultrasound methods in the general population were calculated separately for participants. In this study, for the prevalence of PCOS based on NIH criteria, 15 articles were analyzed showing a prevalence of 8.6% (CI 95%: 5.8–11.4). The lowest prevalence was 3% in the two studies of Hashempur in Isfahan and Rahmanpour in Zanjan which were both on adolescent girls aged 14-18 years old (11, 13).

In total, 16 articles which have investigated the prevalence of PCOS according to the Rotterdam criteria were entered into the statistical analysis. The estimate prevalence of PCOS based on meta-analysis was 19.5% (95% CI: 14.8 – 24.2). The highest prevalence (70%) was for the Mohajerani Tehrani *et al* study (15). The high prevalence in this study could be due to the sample of individuals with gestational diabetes and a mean age of 31.5± 4 years and the least prevalence was for Salehpour *et al* study based on Rotterdam criteria conducted on students with the mean age of 15.84 ±1.05 years (16). One of the criteria for the diagnosis of PCOS based on the Rotterdam criteria is polycystic ovaries on ultrasound in which adolescents have limitations. For instance, vaginal ultrasound provides a better picture of the ovaries than the abdominal ultrasound, especially in obese adolescents, but teens are not allowed to do it (11).

In 4 studies which were used in this meta-analysis, the prevalence of PCOS was 41.4% (95% CI: 14.4 – 68.5) based on ultrasound methods.

In 19 selected articles in this study the prevalence of menstrual dysfunction had been investigated in which their estimated meta-analysis was 28% (95% confidence interval: 21-34). The prevalence of hirsutism had been examined in 14 articles that were entered in the meta-analysis, and the meta-analysis estimate of the prevalence of hirsutism was 13% in this study (95% confidence interval: 9-17). In our study, 16 articles were analyzed to assess the prevalence of acne showing the acne prevalence of 26% (95% CI: 19-33). In addition, 9 articles assessing the prevalence of androgenic alopecia were analyzed which showing the androgenic alopecia prevalence of 9% (95% CI: 6-12). In 5 studies, the prevalence of polycystic ovaries on ultrasound was estimated to be 52% (95% confidence interval: 24-80). Overweight and obesity are symptoms associated with PCOS. For this study, BMI ≥ 25 was considered as overweight. BMI ≥ 30 was defined as obesity and central obesity, or android was defined as waist-hip ratio greater than 0.85 and waist circumference more than 88 cm). In 12 of the involved studies, the prevalence of overweight was investigated and the meta-analysis estimate was 21% (95% confidence interval: 16-25). The prevalence of obesity was noted in 6 of the studies and its meta-analysis estimate was 19% (95% CI: 7-31). Four studies investigated the prevalence of abdominal obesity and the meta-analysis was estimated to be 2% (95% CI: 0-4). In the total of 5 studies, the incidence of infertility was noted with the prevalence of 8% (95% CI: 3-14). According to publication bias figure, the effect of bias in these studies was not significant. In fact, most studies were located inside the Funnel Plot, thus demonstrating that the results of most relevant studies, performed in Iran, were included into the analysis (Figure 4). The following figures show the relationship between the prevalence of PCOS base on NIH and Rotterdam criteria, year of study and sample size due to the Meta regression model. 

In figure 5A the prevalence of PCOS base on NIH has been checked with its year. The negative slope of the Meta regression line (p=0.001) showed that the prevalence of base on PCOS in Iran is rising with a slow slop. In figure 5.B the relation between sample size was compared to the prevalence of PCOS and according to this figure there is a significant relation between the sample size and prevalence (p=0.004).

In figure 6A the prevalence of PCOS base 

on Rotterdam has been checked with its year. The positive slope of the Meta regression line (p=0.82) showed that the prevalence of base on PCOS in Iran is rising with a slow slop, But not statistically significant. In figure 6B the relation between sample size was compared to the prevalence of PCOS, according to this figure there is a significant relation between the sample size and prevalence (p=0.003). In the following figure the circles show the weight of studies and it seems that studies with greater sample sizes are more prevalent and vice versa (Figure 5, 6). 

**Table I:** General data of selected studies in the meta-analysis of the prevalence of PCOS based on criteria National Institute of Child Health and Human Disease of the U.S, Rotterdam and ultrasonic methods

**Author** **(References )**	**City**	**Year**	**Number of the participants**	**Age (Year)** **(mean±SD)**	**Prevalence** **of PCOS** **by NIH criteria** **% (95% CI)**	**Prevalence** **of PCOS** **by Rotterdam** **criteria** **% (95% CI)**	**Prevalence of PCOS by sonography methods** **% (95% CI)**
Asgharnia M (1)	Rasht	2010	1850	17.2 ± 0.07	11.3 (9.9-12.8)		
Mehrabian F (9)	Isfahan	2011	820	24.8 ± 5.1	7 (5.3-8.7)	15.2 (12.7-17.7)	
Salehpour S (16)	Tehran	2010	1430	15.84 ± 1.05		3.42 (2.5-4.4)	
Ramezani Tehrani F (10)		2011	929	34.4 ± 7.6	7.1 (5.4-8.8)	14.6 (12.3-16.9)	
Rahmanpour H (11)	Zanjan	2009	1882	14 - 18	2.9 (2.1-3.7)		
Ramezani Tehrani F (47)	Tehran	2011	1002	29.2 ± 8.7	8.5 (6.8-10.2)		
Zandi S (14)	Kerman	2010	118	22.1 ± 4.2	0 (-0.08-0.08)		48.3 (39.3-57.3)
Ghasemi N (49)	Yazd	2010	332	44.3 ± 3.8		6.6 (2.8-10.4)	
Arefi S (6)	Tehran	2000	720	15.7			42.5 (28.4-56.6)
Mohajeri Tehrani M (15)	Tehran	2009	44	31.5 ± 4	0 (-21.8-21.8)	0 (-0.21-0.21)	
Horri N (55)	Isfahan	2008	157	34.78 ± 6.17	8.3 (4-12.6)		
Mirzaei F (44)	Kerman	2007	92	38.76 ± 5.92	19.5 (11.4-27.6)		
Hashemipour M (13)	Isfahan	2004	1000	14 – 18	3 (1.9-4.1)		
Kalantar Hormozi MR (55)	Shiraz	2007	400	51 ± 10	3.1 (1.4-4.8)		
Naderi T (56)	Shiraz	2011	3190			4.5 (3.8-5.9)	
Noorbala MT (22)	Yazd	2009	97	17.26		0 (-0.09-0.09)	
Dadgostar H (62)	Tehran	2009	71	21.1 ± 4.5		15.5 (7.1-23.9)	
Ghaderi R (51)	Birjand	2004	70	15 – 30		37.1 (21.1-53.1)	
Ghaderi R (52)	Birjand	2004	252		6.3 (2.1-10.5)		
Farnaqy F (48)	Tehran	2002	110	29.7 ± 3.2		49 (39.7-58.3)	
Ansarin H (21)	Tehran	2006	790	20.9		0 (-0.34-0.34)	
Akbari D (12)	Borazjan	2010	200	14 – 48		54.50 (47.6-64.1)	
Akhyany M (53)	Tehran	2006	800	18 – 45	10.62 (8.5-12.8)		
Jebraiili R (57)	Khoramabad	2001					62.5 (51.9-73.1)
Jahanfar S (58)	Tehran	2004	154	14 – 54		16.2 (10.4-22)	
Bani-Hashemi M (59)	Zahedan	2007	180	28.65 ± 9.6		53.30 (46-60.06)	
Kamkhvah AF (61)	Tehran	2008	127		8.9 (3.9-13.9)		
Kashanian M (50)	Tehran	2008	188	28.7 ± 4.6		16 (8.6-23.4)	
Nqash Hosseini SH (60)	Kerman	2003	1000	15 – 18	26.3 (23.6-29)		13.4 (11.3-15.5)
Sajjadi Mirzaei S (46)	Tehran	1996	273	15 – 49		33 (27.4-38.6)	

NIH: National Institute of Child Health and Human Disease of the U.S

Rott: Rotterdam

**Table II:** Prevalence of disorders associated with polycystic ovary syndrome

**Author**	**Prevalence **								
	**PCO**	**Hirsutism**	**Acne**	**Androgenic alopecia**	**Menstrual disorders**	**Overweight**	**Obese**	**Android**	**Infertility**
Asgharnia M (1)		4.91	5.45		20.40				
Mehrabian F (9)		4.02			21.30	19.0	9.0		
Salehpour S (16)		10.70	31.30	3.10			45.0		
Ramezani Tehrani F (10)	16.80	33.80	3.50		18.30				
Rahmanpour H (11)		8.60	11.70	6.90	16.90	8.9		1.4	
Ramezani Tehrani F (47)		25.55	0.30		10.48	30	20.5		
Zandi S (14)	48.3	54.20			37.30	27.3			
Ghasemi N (49)					6.62		1.8		1.80
Mohajeri Tehrani M (15)	65.0	26.0			40.0	85.0		45	
Mirzaei F (44)								19.5	
Hashemipour M (13)		6.0	4.70	3.0	7.40				
Kalantar Hormozi MR(55)		8.90			9.65				
Naderi T (56)		16.40	13.0	5.50		10.3	2.6	0.5	
Noorbala MT (22)		10.80	42.7		14.6				
Ghaderi R (52)			37.10		22.90				
Ghaderi R (51)			22.2			9.5			
Farnaqy F (48)	61.0		32.0	36	38.0	29.0			7.0
Ansarin H (21)			70.0	21.30	38.60	6.50			
Akbari D (12)	70.0		52.50	15.0	35.0	38.0			
Akhyany M (53)		22.80	64.90		38.75	11.0			
Jebraiili R (57)					45.0				3.70
Bani-Hashemi M (59)			25.0	19.40			39.0		13.0
Kashanian M (50)		14.9	9.58	7.46	28.70	12.5			20.20
Nqash Hosseini SH (60									
Sajjadi Mirzaei S (46)					81.30				
Total									

PCO: Poly cystic ovarian

Values were expressed as %.

**Figure 2:**
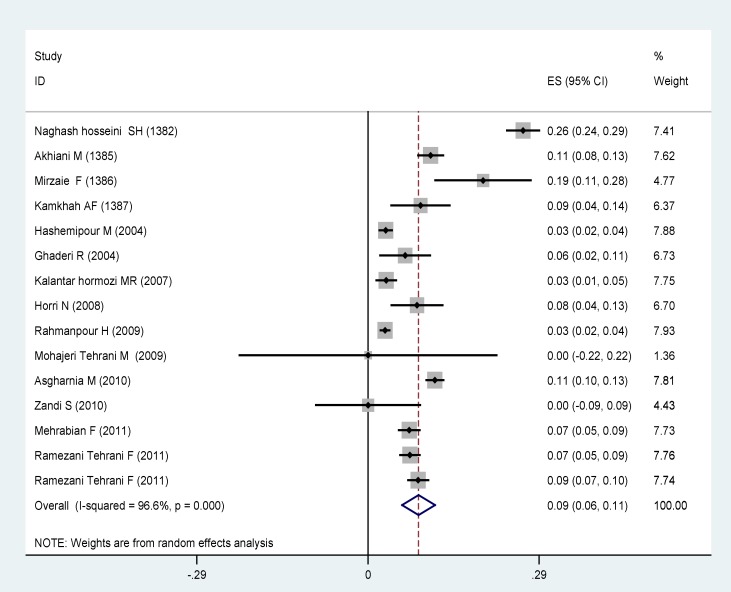
The prevalence of polycystic ovary syndrome based on NIH criteria based on random effects model. Midpoint of each segment is the estimate of prevalence and segment lengths shows the 95% confidence intervals for each study. The diamond mark shows the prevalence in the whole country for all studies

**Figure 3:**
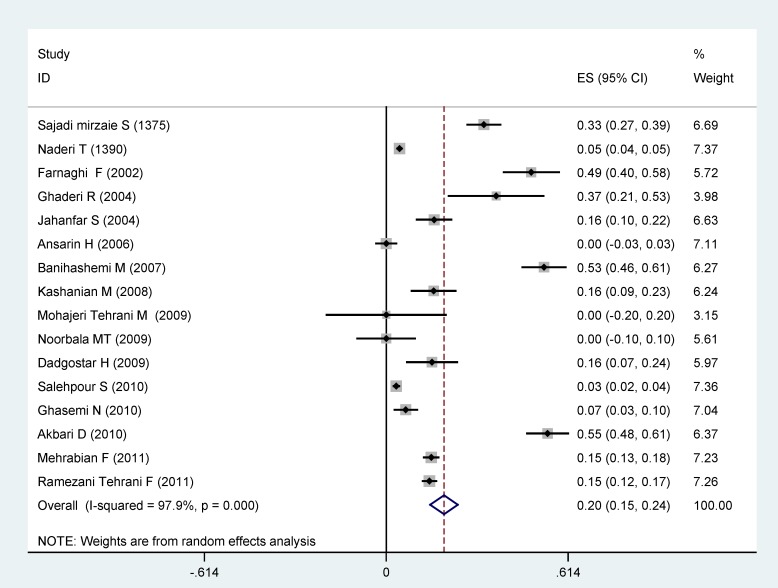
The prevalence of polycystic ovary syndrome based on Rotterdam criteria based on random effects model. Midpoint of each segment is the estimate of prevalence and segment lengths shows the 95% confidence intervals for each study. The diamond mark shows the prevalence in the whole country for all studies

**Figure 4:**
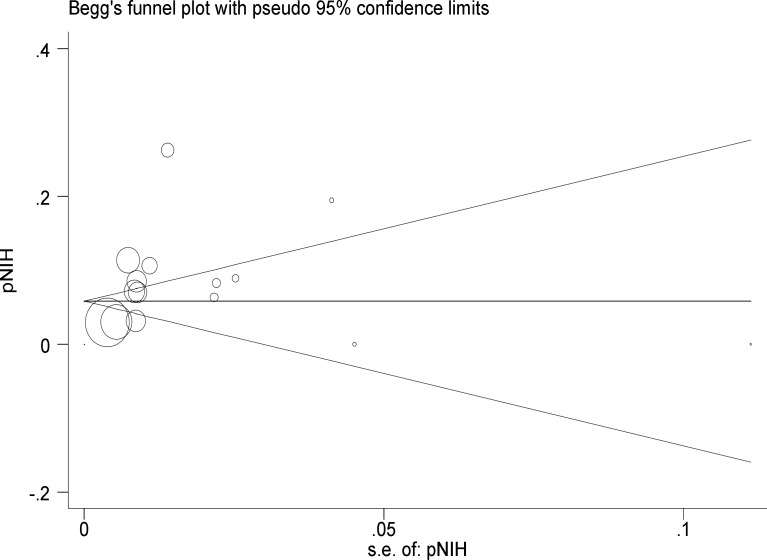
Begg’s funnel plot for publication bias. The diameter of each circle represents the weight in the meta-analysis. Each circle represents the RDs according to the standard error of each RDs. The diameter of each circle represents the weight in the meta-analysis

**Figure 5:**
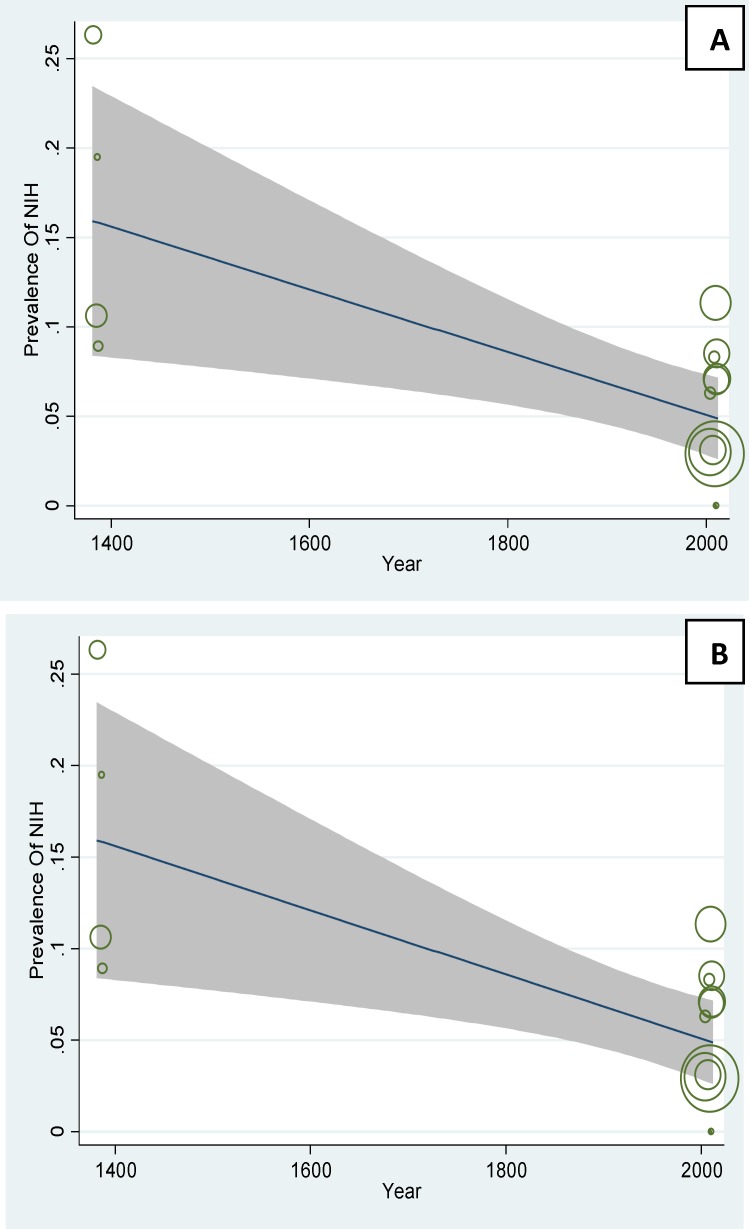
A: The association between prevalence of PCOS based on NIH criteria and year, using Meta regression. B: The association between prevalence of PCOS based on NIH criteria and sample size, using Meta regression

**Figure 6:**
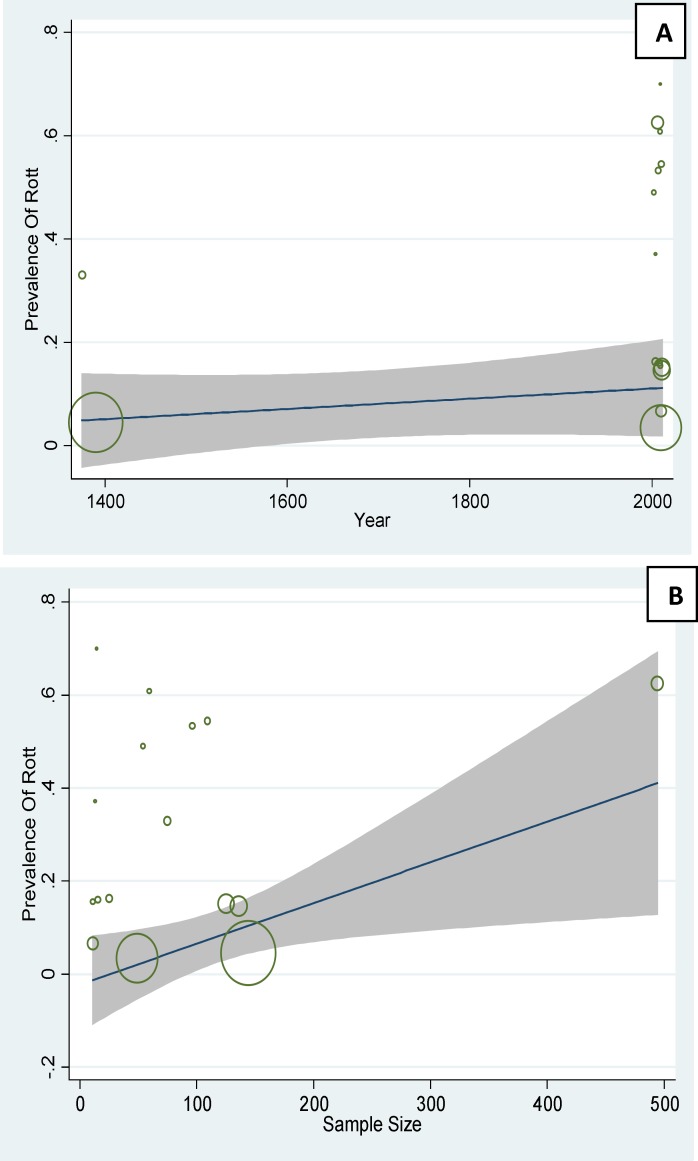
A: The association between prevalence of PCOS based on Rotterdam criteria and year, using Meta regression. B: The association between prevalence of PCOS based on Rotterdam criteria and sample size, using Meta regression

## Discussion

Polycystic ovary syndrome is a heterogeneous disease that is caused by various reasons. In all studies, participants were selected using random selection and no meta-analysis study has been carried out on the prevalence of PCOS in Iran.

From 15 articles found in this study to measure the prevalence of PCOS according to NIH; the prevalence of 12% (95% CI: 9-16) was shown. Diagnosis of PCOS is more difficult in adolescents. It seems that the low prevalence of PCOS in these studies is because of not using ultrasound criteria. The highest prevalence of PCOS based on NIH criteria was 60.2% in Zandi *et al* study in Kerman on women with acne with the mean age of 22.1±4.2 years (14). Various studies show that the prevalence of PCOS is about 10 times more in patients with acne than normal people. Therefore, the final prevalence of PCOS was 9% based on NIH criteria (95% CI: 6-12). The results of this study were in line with the study performed by Diamanti-Kandarakys *et al* on 192 women between 17-45 years old in Greece who have reported the PCOS prevalence of 6.8% based on NIH criteria (17). Also, the prevalence of PCOS was reported to be 6.5% in a study of 145 women between 18-45 years old in Madrid, Spain (18). Kamaraply *et al* reported the prevalence of 6.5% in a population of 3030 people between 15-39 years old (19). Aziz mentioned the PCOS prevalence of 6.6% based on NIH criteria in a study on 400 women between 18-45 years (20). The prevalence of PCOS was also examined according to the Rotterdam criteria. The main criteria for inclusion of studies based on Rotterdam criteria were doing ultrasound. The prevalence of 31% was found from the total 18 studies entered in the analysis (CI 95%: 23-38). Three studies of Mohajerani Tehrani (15) with a prevalence of 70%, Ansarian (21), with a prevalence of 63%, and Noorbala (22) with a prevalence of 61% were excluded due to the distance of their results with the other studies. Therefore, the final prevalence of PCOS based on Rotterdam criteria was estimated to be 20% (95% confidence interval: 15-24). Result of our study was almost similar to that of March in South Australia in which the prevalence of 17.8% was observed among 978 women (23). Lau *et al* showed the PCOS prevalence of 12% among 100 women based on Rotterdam criteria (24). Gati reported the PCOS prevalence of 91% among 109 women in UAE based on Rotterdam criteria (25). In another study, Adams *et al* from England reported the PCOS prevalence of 92% among 350 patients with hirsutism (26). The differences observed between these studies and our study may be related to the higher precision of ultrasound method QUS in western studies. In the west, vaginal ultrasound (vaginal) is done in which the ovaries are more carefully evaluated and there is a greater chance to diagnose polycystic ovary. For this reason, the diagnoses of PCOS have increased nowadays. This method is less possible in Iran due to ethical and practical problems in unmarried women.

The prevalence of PCOS was estimated to be 41% based on ultrasound methods. Farquhar reported the prevalence of PCOS to be 21% in a study of 255 healthy women by ultrasound (27). Prevalence of polycystic ovaries on ultrasound 69% according to Khoury *et al* study (28) and this causes doubt in the use of ultrasound to diagnose PCOS (11).

The prevalence of menstrual disorders in our study was 28%. In a study on 90 women over 19 years with acne, menstrual disorders was observed in 43 patients (48%) (29). According to Khoury *et al* study, oligomenorrhea was seen in 60% of patients (28). Menstrual disorders, particularly oligomenorrhea after the ages of menarche, can be a beginning to ovulatory dysfunction and infertility, and complications resulting from abnormal increase in estrogens and androgens in later years. In fact, the complications of PCOS can be prevented to some extent by early diagnosis (6).

In our study, the prevalence of polycystic ovaries on ultrasound was 52% which was in line with that study by Van Hoof *et al* (2000). This study showed that in oligomenorrhoea people, PCO signs can be seen on ultrasound in 45% of cases (30). Michelmore *et al* also reported the PCO prevalence of 33% in adolescent girls (31).

The prevalence of infertility was estimated as 0.08% in this study. One of the major causes of infertility is ovarian dysfunction which covers 30 to 40% of infertility cases. PCOS is important since it affects fertility and is the most common cause of ovarian dysfunction (32). In addition to the higher prevalence of infertility in patients with PCOS due the incidence of hyperandrogenism and increase in resistance to insulin, the risk of spontaneous abortion increases in these patients in case of pregnancy (33). Glueck studied 72 patients with PCOS during pregnancy and reported 62 abortions (62%) in 100 pregnancies (34).

According to statistics, the prevalence of hirsutism was reported to be 25-30% in the total population. The meta-analysis estimate in this study was 17% from the total 15 reviewed articles (95% confidence interval: 12-21). Zandi's study was excluded due to the distance of its high incidence with other studies. As a result, the final prevalence of hirsutism was 13% in this study (95% confidence interval: 9-17) (14). The result of our study is almost the same as Harrison’s study with the prevalence of 10% and Mcknight’s study with the prevalence of 9% (35, 36). Hirsutism incidence varied in different parts of the world from 3% in Japanese women to 70% in Caucasians women (37, 38). The differences observed in the prevalence of national and international studies are possibly due to racial differences and differences between age groups of the sample. In our study, the prevalence of acne was 26% that was similar to the study of Cibula *et al* which had reported the incidence of moderate acne to be 22% (29). Kilkenny *et al* (1998) reported the incidence of moderate to severe acne to be 17% (39). In a study, acne in women with PCOS and control group women was reported to be 83% and 19%, respectively. Approximately 80% of women with severe acne, experience increase in blood androgen levels (40). As a result, androgenic hormone levels were higher in patients with severe acne and severe acne has a significant relationship with the incidence of PCOS.

Androgenic alopecia or male pattern baldness is one of the other hyper androgenic symptoms. Compiled the prevalence of androgenic alopecia was estimated to be 11% from the total of 9 papers analyzed (95% confidence interval (CI 95%: 8-14). The study of Farnaqy was excluded due to a high distance of its results from other studies. Therefore, the final prevalence was 9%. These results were similar to the study of 20-30 years old Caucasus women which reported the prevalence of androgenetic alopecia to be 6 to 12% (41). In another study, 40% of men and 30% of women showed androgenetic alopecia until 40 years old (42). The difference in results may be related to several factors. These factors are age and increasing androgens in menopausal women, diet in terms of iron, other minerals and protein composition, exposure to different types of stress, stressful life, a negative perception of body shape and adjustment disorders as well as genetics effects. Hormonal disorders, especially thyroid dysfunction and other hormonal diseases cause excessive androgens secretion, and thus have a significant role in hair loss. 

PCOS is not only an endocrine- reproduction disease, but also is a metabolic disorder. Obesity is a consistent feature of PCOS seen in 40-50% of patients and has an increasing and synergistic effect in the emergence of PCOS and exacerbates the existing endocrine disorders (43). In our study, the prevalence of overweight women was 21%. The prevalence of obesity was also estimated to be 19%. Conway *et al* (1989) observed obesity in 35-60% of patients (43). The prevalence of BMI over 30 was higher in patients with PCOS than in others, while some other studies did not show this result. These findings suggest significant racial differences in the prevalence of overweight women in PCOS syndrome (20). It seems that increase in PCOS symptoms with age increase is associated with accumulation of adipose tissue. About 1:3 obese patients experience PCOS and impaired glucose tolerance and 7.5 to 10% experience type II diabetes. Android central obesity is presented as a risk factor for PCOS which increases the probability of insulin resistance and type II diabetes (45). In this study, the prevalence of obesity was estimated to be 20.5%. Mor et al compared 32 women with PCOS and insulin resistance with 46 women with this syndrome without insulin resistance and showed that the relationship between insulin resistance and body mass index was significant (45). Since insulin resistance is a key factor in the incidence of type II diabetes, it is suggested that women with PCOS are at a higher risk for type II diabetes (6).

The analysis of incidence wasn’t calculated based on AES criteria, because few studies investigated the prevalence of PCOS based on these criteria. PCOS prevalence in different studies shows different results which was a limitation for carrying out this study. The observed differences in the prevalence of PCOS in various studies were due to different sampling methods and different measured parameters. For example, the prevalence in adolescents is different from the prevalence in women of childbearing age. Another reason for the observed differences in the prevalence of PCOS is the presence of different criteria for its definition. Many studies have not indicated the criteria type; therefore, another limitation of this study was the lack of a single definition in all studies. Lack of access to full text articles and even abstracts related to this topic in some cases is another limitation of this study. The strength points of this study are: prevalence was mentioned based on different criteria, and Sampling is done Randomize.

## Conclusion

The prevalence of PCOS is not high in Iran, but it appears that increasing the intensity of PCOS symptoms with increasing age is associated with accumulation of adipose tissue.

Menstrual dysfunction is the most common complication index associated with PCOS in Iranian women which can lead to infertility in the long term. Acne, obesity, hirsutism and alopecia are the next most common factors, respectively. Due to some differences in the way people live in different communities, various results are obtained on the relationship between environmental factors and the prevalence of PCOS. However, all studies agree on the relationship between clinical symptoms and the prevalence of PCOS and the results of our study also focus on this. According to the findings, and the risks of other complications such as cardiovascular disease and infertility, prevention of PCOS is important. As a result, health authorities should offer plans in this respect to the community.
